# Sedimentation Yields Long-Term Stable Protein Samples as Shown by Solid-State NMR

**DOI:** 10.3389/fmolb.2020.00017

**Published:** 2020-02-21

**Authors:** Thomas Wiegand, Denis Lacabanne, Anahit Torosyan, Julien Boudet, Riccardo Cadalbert, Frédéric H.-T. Allain, Beat H. Meier, Anja Böckmann

**Affiliations:** ^1^Physical Chemistry, ETH Zürich, Zurich, Switzerland; ^2^Institute of Molecular Biology and Biophysics, ETH Zürich, Zurich, Switzerland; ^3^Molecular Microbiology and Structural Biochemistry, UMR 5086 CNRS/Université de Lyon, Labex Ecofect, Lyon, France

**Keywords:** solid-state NMR, sedimentation, stability, proteins, nucleotides

## Abstract

Today, the sedimentation of proteins into a magic-angle spinning (MAS) rotor gives access to fast and reliable sample preparation for solid-state Nuclear Magnetic Resonance (NMR), and this has allowed for the investigation of a variety of non-crystalline protein samples. High protein concentrations on the order of 400 mg/mL can be achieved, meaning that around 50–60% of the NMR rotor content is protein; the rest is a buffer solution, which includes counter ions to compensate for the charge of the protein. We have demonstrated herein the long-term stability of four sedimented proteins and complexes thereof with nucleotides, comprising a bacterial DnaB helicase, an ABC transporter, an archaeal primase, and an RNA polymerase subunit. Solid-state NMR spectra recorded directly after sample filling and up to 5 years later indicated no spectral differences and no loss in signal intensity, allowing us to conclude that protein sediments in the rotor can be stable over many years. We have illustrated, using an example of an ABC transporter, that not only the structure is maintained, but that the protein is still functional after long-term storage in the sedimented state.

## Introduction

Using solid-state Nuclear Magnetic Resonance (NMR), a wide variety of biological materials can be studied, such as micro- or nanocrystals, fibrillar aggregates, and proteins embedded in membranes. Sedimentation of dissolved proteins from the solution state directly into the magic-angle spinning (MAS) rotor has lately become the most important sample preparation approach for solid-state NMR (Bertini et al., 2011, 2012, 2013; Gardiennet et al., 2012; Ravera, 2014; van der Wel, 2018; Lacabanne et al., 2019a). This can be achieved in an ultracentrifuge (Böckmann et al., 2009; Bertini et al., 2012; Gardiennet et al., 2012) using specially designed rotor-filling tools (Böckmann et al., 2009; Mandal et al., 2017). With this approach, typical protein concentrations on the order of 400 mg/mL were achieved using the NMR rotor (Wiegand et al., 2016a) in which the protein was still hydrated and typically had around 50 weight percentages of water (Bertini et al., 2013). No long-range order in such sediments was observed by X-ray diffraction (Gardiennet et al., 2012). Sample preparation by sedimentation allowed for the investigation of proteins that are difficult (or even impossible) to crystallize. Theoretically, the molecular mass of the protein determines the success of sedimentation, and proteins, such as the RNA polymerase subunits Rpo4/7^∗^ (the ^∗^ indicates that the Rpo7 unit is uniformly ^13^C/^15^N labeled) with a molecular weight of 34 kDa (Torosyan et al., 2019), the pRN1 primase with 40 kDa (Boudet et al., 2019), the neonatal Fc receptor with 40 kDa (Stöppler et al., 2018) as well as the human superoxide dismutase with 32 kDa (Fragai et al., 2013), the bacterial helicase DnaB with 708 kDa (Gardiennet et al., 2012), the iron-storage protein ferritin with 480 kDa (Bertini et al., 2012), a variety of supramolecular assemblies (Lecoq et al., 2018; Gauto et al., 2019), and PEGylated proteins (Ravera et al., 2016), were shown to form sediments suitable for solid-state NMR. This is for some of these systems probably a consequence of oligomerization that has been induced by the high protein concentrations (Bertini et al., 2013; Fragai et al., 2013). Protein–protein complexes were studied by co-sedimentation (Gardiennet et al., 2016; Xiang et al., 2018) and protein–ligand complexes by sedimentation with the respective ligand (e.g. nucleic acids) directly into the NMR rotor (Wiegand et al., 2016a; Kaur et al., 2018; Stöppler et al., 2018; Lacabanne et al., 2019b).

Multiple 3D and/or 4D correlation experiments are usually collected to assign protein resonances. A basic set of 3D experiments requires, for larger proteins, around 1 month of solid-state NMR measurement time [e.g. 46 days for the 110 amino acids AL-09 VL immunoglobulin light chain fibrils (Piehl et al., 2017), 41 days for the 153 amino acids N-terminal domain of *Hp*DnaB (Wiegand et al., 2016b), 42 days for the 350 amino acids TET2 (Gauto et al., 2019), and 29 days for the 488 amino acids DnaB (Wiegand et al., 2019)]. It is beneficial if the 3D set is recorded on the same sample. Consequently, the sample has to be stable over months for recording multidimensional solid-state NMR experiments.

The stability of a protein sample can in principle be affected by several factors, such as chemical processes (e.g. oxidation, deamination, and hydrolysis) (Celi and Gabai, 2015; Davies, 2016), temperature (Querol et al., 1996; Vogt et al., 1997; Bischof and He, 2006), pressure (Silva and Weber, 1993; Gross and Jaenicke, 1994; Hillson et al., 1999; de Oliveira and Silva, 2015), and freeze–thaw cycles (Manning et al., 1989, 2010). One of the major sources of protein instabilities induced by chemical processes involves oxidation of the protein, mainly at the side chains, such as thiol oxidation (cysteine and methionine), aromatic hydroxylation (tryptophane and tyrosine), deamination (lysine, arginine or backbone), and the formation of carbonyl groups (Celi and Gabai, 2015; Davies, 2016). The oxidation process is catalyzed by free radicals, radiation (high energy or UV-visible light), and also traces of metal ions (e.g. Fe^3+^ and Cu^2+^) (Zhang et al., 2013; Davies, 2016). Solvent-exposed cysteines can form intermolecular disulfide bonds, which can be a source of aggregation (Manning et al., 2010). Macromolecular crowding has also been discussed in the context of protein stability in the sense that a high concentration of a crowding reagent decreases the available space for the protein of interest (excluded volume effect), which favors the native protein state in certain cases (Minton, 1981; Wang et al., 2012). A further parameter that has been identified to influence the NMR spectral quality of proteins is the loss of water during a MAS NMR experiment (e.g. due to an incompletely sealed rotor cap), which results in broadened NMR resonances (Fragai et al., 2013).

Here, we describe our experimental observations regarding the stability of four sedimented samples from different protein systems: (i) the bacterial helicase DnaB from *Helicobacter pylori* in a complex with ADP:AlF_4_^–^ and DNA (Wiegand et al., 2019), (ii) the archaeal pRN1 primase from *Sulfolobus islandicus* in a complex with DNA and ATP (Boudet et al., 2019), (iii) the membrane protein BmrA, an ABC transporter from *Bacillus subtilis* (Lacabanne et al., 2019b), and (iv) the two RNA polymerase subunits Rpo4/7^∗^ from *Methanocaldococcus jannaschii*. We use 2D DARR spectra and ^31^P cross polarization (CP) MAS experiments to assess the long-term stability of these sedimented protein samples. We show that the sedimented samples investigated are stable over several months to years in an NMR rotor.

## Results and Discussion

**FIGURE 1:**
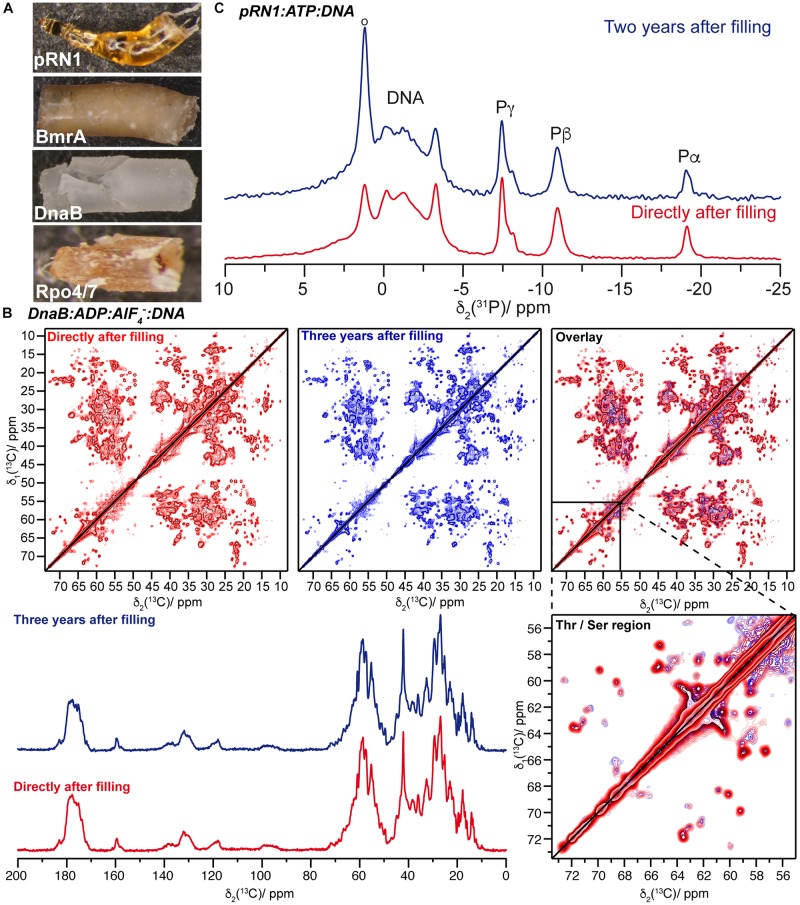
**(A)** Protein sediments drilled out of solid-state NMR rotors of Rpo4/7^∗^, DnaB, BmrA, and the pRN1 primase (bottom to top). These sediments belong to different protein samples and not systematically to the ones for which spectra are reported herein. **(B)** The top panel shows the comparison of 20 ms ^13^C-^13^C DARR correlation spectra of DnaB:ADP:AlF_4_^–^:DNA recorded directly after rotor filling (red, taken from reference Wiegand et al., 2019) and 3 years later (blue). The bottom panel presents a ^13^C,^1^H 1D CP MAS spectra recorded on DnaB:ADP:AlF_4_^–^:DNA. **(C)** ^31^P,^1^H CPMAS spectra of the quaternary pRN1:ATP:ATP:DNA complex collected directly after sample filling [red, adapted from reference (Boudet et al., 2019)] and 2 years later (blue).

Solid-state NMR protein samples were sedimented into the NMR rotors in an ultracentrifuge (typically overnight at 200,000 × *g*) using a home-made rotor-filling tool (Böckmann et al., 2009). Such tools withstand the high centrifugal forces during filling, which tend to press the rotor in the filling tool material (Lacabanne et al., 2019a). Figure 1A shows protein samples of the pRN1 primase, the ABC transporter BmrA, the DnaB helicase, and the RNA polymerase subunit Rpo4/7^∗^ which were drilled out of solid-state NMR rotors. These samples were stored for several years in the rotors after filling. The samples possess a gel-type appearance and are quite densely packed, as expected.

We have recorded ^13^C-^13^C 20 ms Dipolar Assisted Rotational Resonance (DARR) (Takegoshi et al., 1999, 2001) fingerprint spectra on the proteins and complexes thereof. In all cases, one spectrum was recorded directly after filling the NMR rotor and the second one on the same protein sample several years later. In the meantime, the samples were stored at 4°C or −20°C (for pRN1) and were also frequently used for further NMR experiments.

The example of the DnaB:ADP:AlF_4_^–^:DNA complex, a hexamer in a complex with six ADP:AlF_4_^–^ molecules and one DNA molecule, is shown in Figure 1B. The sample reveals nearly similar NMR spectra (linewidths, positions, and intensities – see Supplementary Figure S1 for the difference of the two DARR spectra and Supplementary Figure S2 for a peak-by-peak comparison of spectral features) indicating the long-term structural and conformational stability of the sample. Together with the nearly identical intensities of 1D ^13^C,^1^H CP-spectra (Figure 1B), we can conclude that almost no sample degradation took place.

**FIGURE 2:**
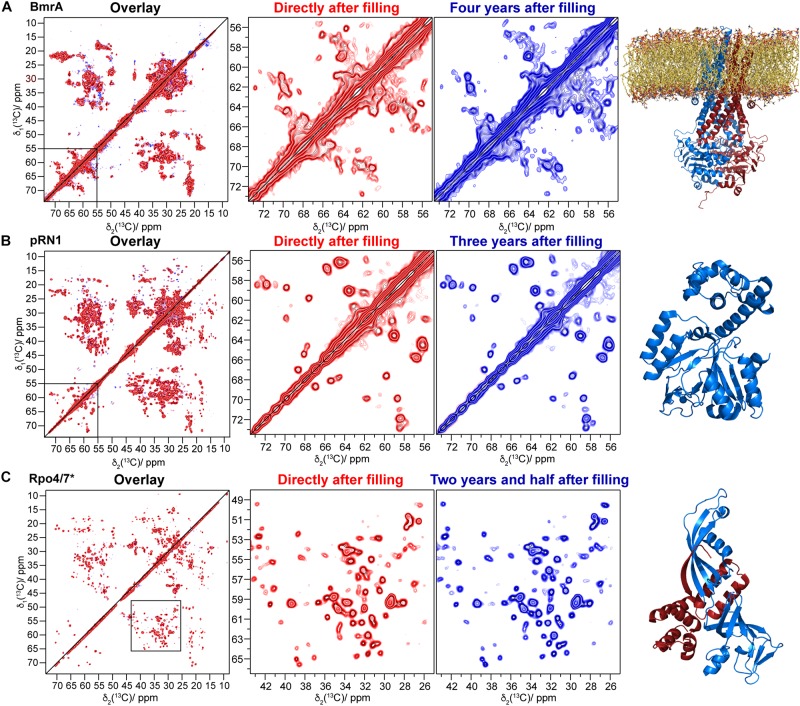
Comparison of 20 ms ^13^C-^13^C DARR correlation spectra recorded directly after filling (red) and up to several years later (blue): **(A)** BmrA E504 [a homology model based on SAV1866 is shown in the last column, PDB ID: 2HYD (Dawson and Locher, 2006), the red spectrum is taken from reference (Lacabanne et al., 2019b)], **(B)** the pRN1 primase (PDB ID: 6GVT), and **(C)** ^13^C/^15^N labeled Rpo7 (PDB ID: 1GO3, blue ribbons) in complex with unlabeled Rpo4 [red ribbons, where the red spectrum is taken from reference (Torosyan et al., 2019)]. The corresponding protein structures are shown on the right.

**FIGURE 3:**
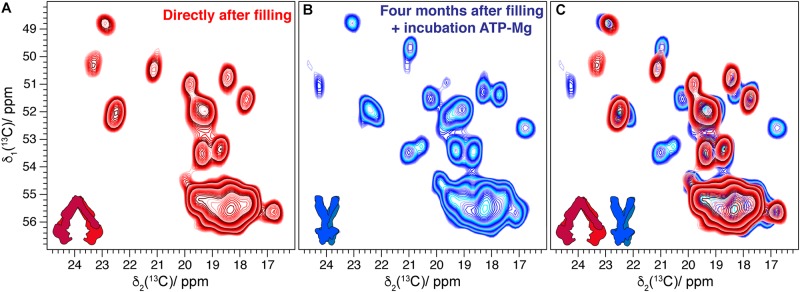
**(A)** Comparison of alanine region spectral fingerprints of 20 ms ^13^C-^13^C DARR correlation spectra of BmrA-E504A in its lipids environment recorded directly after rotor filling [taken from reference (Lacabanne et al., 2019b)]. **(B)** DARR spectrum collected on the same sample resuspended in buffer after being stored for 4 months, incubated with ATP-Mg, and sedimented again in the rotor. **(C)** Overlay indicating CSPs and new peaks corresponding to the conformational changes of the transporter [according to reference (Lacabanne et al., 2019b)].

BmrA-E504A is a non-catalytic mutant in which the ATP hydrolysis is abolished. Nevertheless, this variant can still operate conformational changes that are induced by ATP binding (Orelle et al., 2008). After 4 years of storage in the NMR rotor, the sample of the membrane protein BmrA-E504A in absence of ligand showed the same DARR spectrum with the same signal-to-noise ratio compared to the one measured directly after rotor filling [Figure 2. Note that LVIKH were selectively unlabeled to reduce spectral overlap in the spectrum (Lacabanne et al., 2018, 2019b)]. An interesting question is whether an aged sedimented sample can still undergo conformational changes, such as those previously detected by solid-state NMR reflecting the transition between the inward- and outward-facing state of the transporter (Lacabanne et al., 2019b). Therefore, a 4-month-old rotor containing the mutant BmrA-E504A apo (in its inward-facing state, see Figure 3A for the spectrum) was opened, and the sediment was unpacked, resuspended in the presence of ATP-Mg, and sedimented a second time into the solid-state NMR rotor. The new spectrum (Figure 3B, for an overlay with the spectrum obtained directly after rotor filling see Figure 3C) indeed indicated the conformational change to the outward-facing form as described previously by Lacabanne et al. (2019b).

For the pRN1 archaeal primase in complex with ATP and DNA (Boudet et al., 2019), we used ^31^P,^1^H cross-polarization spectra to probe whether the nucleotides remain bound to the enzyme after 2 years (Figure 1C). Such cross-polarization-based spectra only show immobilized ATP and DNA molecules, whereas such species dissolved in the supernatant are filtered out (Wiegand et al., 2016a); however, this could be detected by solution-state-like INEPT experiments (Wiegand et al., 2016a). The spectra reveal that ATP remains bound to the protein in its non-hydrolyzed form. In its active conformation, two ATP molecules should bind to one functional pRN1 primase (Boudet et al., 2015). This assumption is supported by the splitting of the ATP Pγ-resonance (Boudet et al., 2019). This allows us to conclude that the pRN1:ATP:DNA complex is stable over a time span of more than 2 years, although free ATP is not available in the rotor anymore due to ATP autohydrolysis. The hydrolysis of ATP also most likely explains the increasing resonance overtime at around 1.5 ppm, which is due to its typical ^31^P chemical shift toward a phosphate group assigned to some phosphate species that coordinate to the protein in an unspecified manner (resonance highlighted by a circle in Figure 1C, top panel). The pRN1 sample was stored at −20°C after the rotor filling. After 3 years, the 20 ms ^13^C-^13^C DARR spectrum is almost identical compared to the spectrum recorded directly after the rotor filling (Figure 2B). More specifically, no peak doubling indicating the formation of a second ATP- or DNA-free population is detected. The same conclusions can be drawn from the spectra of the Rpo4/7^∗^ RNA polymerase subunit for which the DARR spectra directly recorded after the rotor filling and 2.5 years later are highly similar (see Figure 2C).

The experimental observation of stable protein sediments over many years might be related to several precautions. To avoid cysteine oxidation of the protein sample, a sufficient amount of the redox reagent dithiothreitol (DTT) is typically added to the protein sample before sedimentation (except if the protein contains disulfide-bonds). Moreover, to avoid bacterial or fungal contamination, each rotor also contains a sufficient amount of sodium azide [0.01% (m/v)] (Snyder and Lichstein, 1940; Bowler et al., 2006). Depending on the time between protein–ligand complex formation and rotor filling, residual DTT might still be present in the rotor. Regarding the importance of oxidative processes, we estimated the amount of oxygen solubilized in the buffer within the NMR rotor, assuming that the rotor was sealed appropriately. This resulted in around 6 nmol of O_2_ in a 3.2 mm rotor that is ∼2 orders of magnitude less than the amount of protein. This estimation is based on a solubility of O_2_ of ∼8.5 mg/L at a O_2_ partial pressure of 0.21 atm (Rettich et al., 2000; Miyamoto et al., 2014), and the assumption that approximately half of the rotor is filled with the buffer (23 μL). A compelling reason for the absence of oxidation seems thus to be the low oxygen concentration compared to the high concentration of protein. As a higher temperature leads to faster denaturation of proteins, NMR measurements are typically performed at 5°C (sample temperature) (Böckmann et al., 2009), and the NMR rotor is stored at this temperature. An additional parameter altering protein stability is pressure. Indeed, many studies showed that high pressure induces dissociation and denaturation of proteins (Hillson et al., 1999; de Oliveira and Silva, 2015). However, the maximal centrifugal pressure in an NMR rotor acts on the ZrO_2_ rotor wall during MAS (∼1,500 bar at 17.0 kHz MAS in a 3.2 mm rotor, F. Engelke, lecture notes of EBSA solid-state NMR school 2014). The pressure value acting on the protein is even lower and is estimated between 60 and 91 bars (Kawamura et al., 2007; Asano et al., 2012). Consequently no pressure-induced protein degradation is expected (where this would, for example, occur at the 2,500 bar for ubiquitin (Charlier et al., 2018), 3,000 bar for PHS SNase (Roche et al., 2012), or 1,000 bar for spinach photosystem II (Tan et al., 2005)).

Our results on four sedimented protein samples show that proteins, protein–protein complexes, and protein–ligand complexes remain stable for years once sedimented into an NMR rotor. In the case of BmrA, the protein even retains its ability to transform into the outward-facing conformation upon addition of ATP. Our results are definitely of practical importance for solid-state NMR spectroscopists and participate in understanding better the properties of sedimented protein samples.

## Materials and Methods

### Solid-State NMR Experiments

^13^C-detected solid-state NMR spectra were acquired at 20.0 T static magnetic-field strength using a 3.2 mm Bruker Biospin E-free probe (Gor’kov et al., 2007). The MAS frequency was set to 17.0 kHz. The 2D spectra were processed with the software TOPSPIN (version 3.5, Bruker Biospin) with a shifted (3.0) squared cosine apodization function and automated baseline correction in the indirect and direct dimensions. The sample temperature was set to 278 K (Böckmann et al., 2009). All spectra were analyzed with the software CcpNmr (Fogh et al., 2002; Vranken et al., 2005; Stevens et al., 2011) and referenced to 4,4-dimethyl-4-silapentane-1-sulfonic acid (DSS). Typical CP-conditions for all experiments described herein were ω(^1^H) = 60 kHz and ω(^13^C) = 45–49 kHz. 90 kHz SPINAL-64 ^1^H decoupling (Fung et al., 2000) was applied during evolution and detection. Typical acquisition times in the direct dimension were 15.4 ms and, in the indirect dimension, 12.8 ms. ^31^P-detected experiments were acquired at 11.7 T in a Bruker 3.2 mm probe using a spinning frequency of 17.0 kHz. The spectra were referenced to 85% H_3_PO_4_. The CP conditions were ω(^1^H) = 60 kHz and ω(^31^P) = 45 kHz. 90 kHz SPINAL-64 ^1^H decoupling was applied during detection.

### Protein Sample Preparation

The preparations of the *Hp*DnaB:ADP:AlF_4_^–^:DNA (Wiegand et al., 2019), the BmrA mutant E504A (Lacabanne et al., 2019b), pRN1:ATP:DNA (Boudet et al., 2019), and Rpo4/7^∗^ (only Rpo7 is uniformly labeled) (Torosyan et al., 2019) were prepared as described previously. In short, the proteins were recombinantly expressed in presence of ^13^C-glucose (2 g/L) and ^15^N-ammonium chloride (2 g/L) as sole sources of carbon-13 and nitrogen-15, respectively (for Rpo4/7^∗^ only Rpo7 was expressed in minimum medium supplemented with ^13^C-glucose and ^15^N-ammonium chloride).

#### *Hp*DnaB

The purification of *Hp*DnaB was achieved by a heparin-agarose affinity chromatography followed by an anion exchange chromatography with 2.5 mM sodium phosphate pH 7.5 and 130 mM NaCl. The complex formation with ADP:AlF_4_^–^ and single-stranded DNA (20 thymidine nucleotides) is described in reference (Wiegand et al., 2019).

#### BmrA

Purification of BmrA-E504A was performed with a Ni-NTA purification system using a gravity column. The eluted protein was desalted using dedicated columns (PD10 – GE Healthcare Life Sciences) with 50 mM Tris–HCl pH 8.0, 100 mM NaCl, 10% glycerol, and 0.2% DDM (m/v). *Bacillus subtilis* lipids destabilized with Triton X-100 were added to the protein. The quantity of lipids follows a lipid-to-protein ratio of 0.5 (m/m). The DDM and Triton X-100 were removed using dialysis with Bio-beads (BioRad) for 9 days. The proteolipisomes were collected by centrifugation. LVIKH were selectively unlabeled, as described in references (Lacabanne et al., 2018, 2019b).

#### Rpo4/7^∗^

For the complex Rpo4/7, both proteins were expressed and purified separately, as described previously (Torosyan et al., 2019). In brief, Rpo4 was purified as a GST-fusion protein using glutathione agarose affinity chromatography in a P300 buffer (20 mM Tris/acetate pH 7.9, 300 mM potassium acetate, 10 mM magnesium acetate, 0.1 mM zinc sulfate, 5 mM DTT, and 10% glycerol) and eluted by P300 supplemented with 10 mM glutathione. The GST-tag was cleaved off by overnight incubation with thrombin at 37°C and subsequently removed by a 20-min heat shock of the cleaved elution fractions at 65°C followed by centrifugation.

The purification of uniformly ^13^C and ^15^N-labeled Rpo7^∗^ was carried out from inclusion bodies that were, after multiple rounds of washing, resuspended in 20 mM Tris/acetate, pH 7.9, 100 mM potassium acetate, 0.1 mM zinc sulfate, 10 mM magnesium acetate, 10% glycerol, and 6 M urea. The urea-soluble fraction containing Rpo7 was combined with Rpo4, and the complex was formed via urea-based unfolding-refolding dialysis using a slight molar excess of Rpo7^∗^ (1:1.2). Heat shock and centrifugation (as above) were used to remove incorrectly folded Rpo4/7^∗^ complexes as well as remaining sole Rpo7^∗^, yielding pure, heat-stable Rpo4/7^∗^ in the supernatant.

#### pRN1 Primase

The pRN1 primase was purified with an Ni-NTA purification system. The fractions containing the enzyme were dialyzed overnight with thrombin to remove the His-tag. The dialyzed protein was concentrated and loaded on a gel-filtration column with 25 mM Na_2_HPO_4_/NaH_2_PO_4_, NaCl 50 mM, and a pH of 7.

All proteins were concentrated up to 30 mg/mL by centrifugation and sedimented directly into the solid-state NMR rotor in an ultracentrifuge (overnight at 200,000 × *g*) using a homemade rotor filling tool (Böckmann et al., 2009).

## Data Availability Statement

All datasets generated for this study are included in the article/Supplementary Material.

## Author Contributions

DL, AT, JB, and RC prepared the samples. TW and DL performed the NMR experiments, analyzed the data, and wrote the manuscript with input from all authors. TW, DL, FA, BM, and AB designed and supervised the research. All authors approved the submitted version of the manuscript.

## Conflict of Interest

The authors declare that the research was conducted in the absence of any commercial or financial relationships that could be construed as a potential conflict of interest.
