# Supplementary material for: Sedimentation Yields Long-Term Stable Protein Samples as Shown by Solid-State NMR
Source: Front Mol Biosci. 2020 Feb 21;7:17. doi: 10.3389/fmolb.2020.00017 (PMC7047159; doi:10.3389/fmolb.2020.00017)
Supplement: Supplementary file 1 [file Data_Sheet_1.docx]

**Supplementary Information for**

**Sedimentation yields long-term stable protein samples as shown by solid-state NMR**

Thomas Wiegand^a,#,*^, Denis Lacabanne^a,#^, Anahit Torosyan^a^, Julien Boudet^b^, Riccardo Cadalbert^a^, Frédéric H.-T. Allain^b^, Beat H. Meier^a^ and Anja Böckmann^c^

*^a^ Physical Chemistry, ETH Zurich, 8093 Zurich, Switzerland*

^b^ *Institute of Molecular Biology and Biophysics, ETH Zurich, 8093 Zurich, Switzerland*

*^c^* *Molecular Microbiology and Structural Biochemistry UMR 5086 CNRS/Université de Lyon, Labex Ecofect, 69367 Lyon, France*

*# : Both authors contributed equally.*

^*^Corresponding author: thomas.wiegand@nmr.phys.chem.ethz.ch


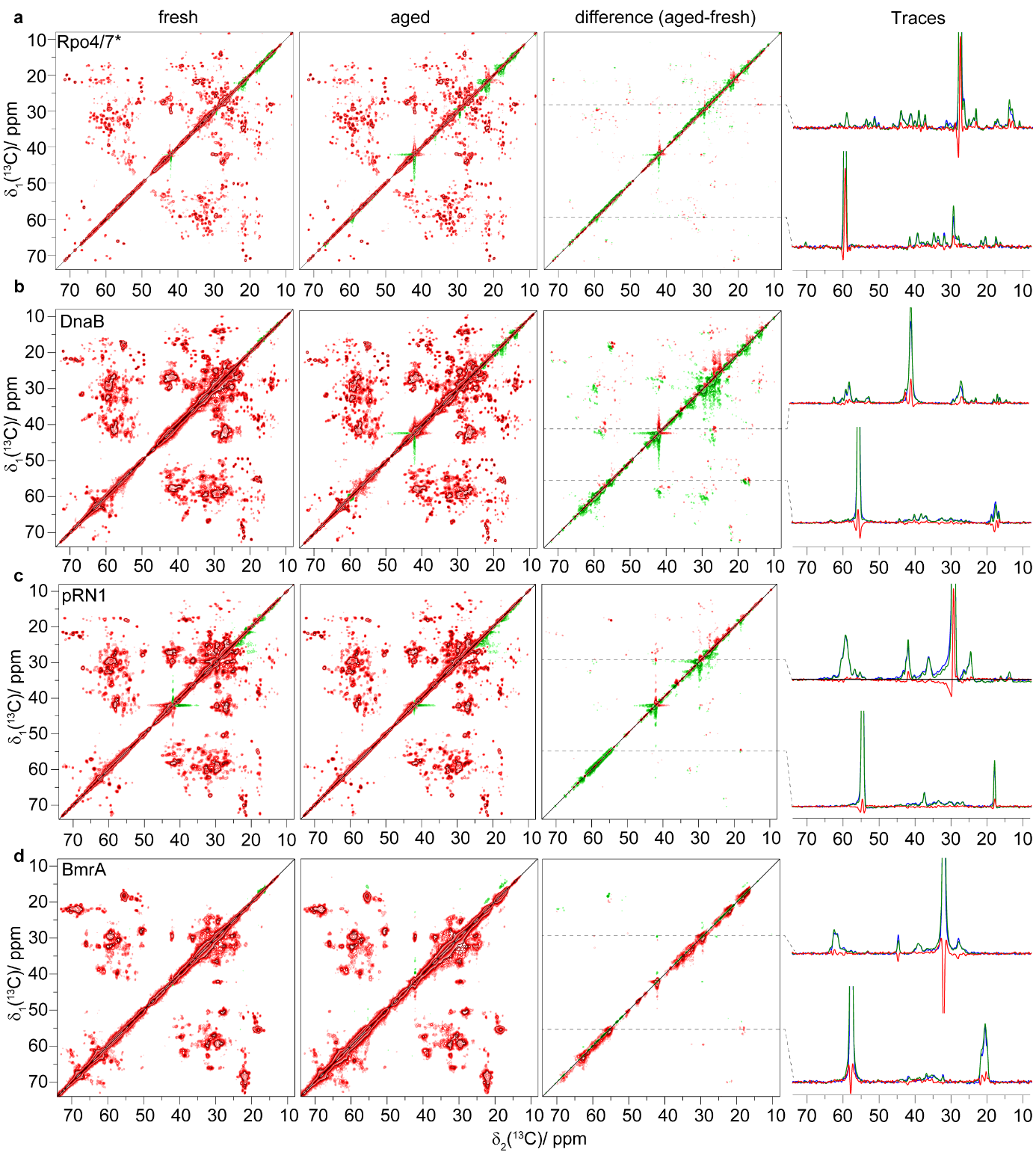


**Figure S1**: *Difference spectra of 20 ms ^13^C-^13^C DARR correlation spectra for fresh and aged samples of the four proteins studied.* The same number of scans has been used for the spectra recorded directly after filling (denoted as fresh) and after a certain period of time (denoted as aged, see main text for the time spans). The last column shows two representative 1D traces for each 2D spectrum along F2 (indicated by dashed lines in the 2D spectrum) in which the most significant differences have been observed. The spectra are shown on the same scale (blue: fresh sample, green: aged sample and red: difference spectrum). Differences in the spectra are within the experimental uncertainty, e.g. due to slightly different experimental settings, changes over time in the experimental setup (i.e. probe efficiency), differences in magnet shims and/or differences in sample temperatures.

**
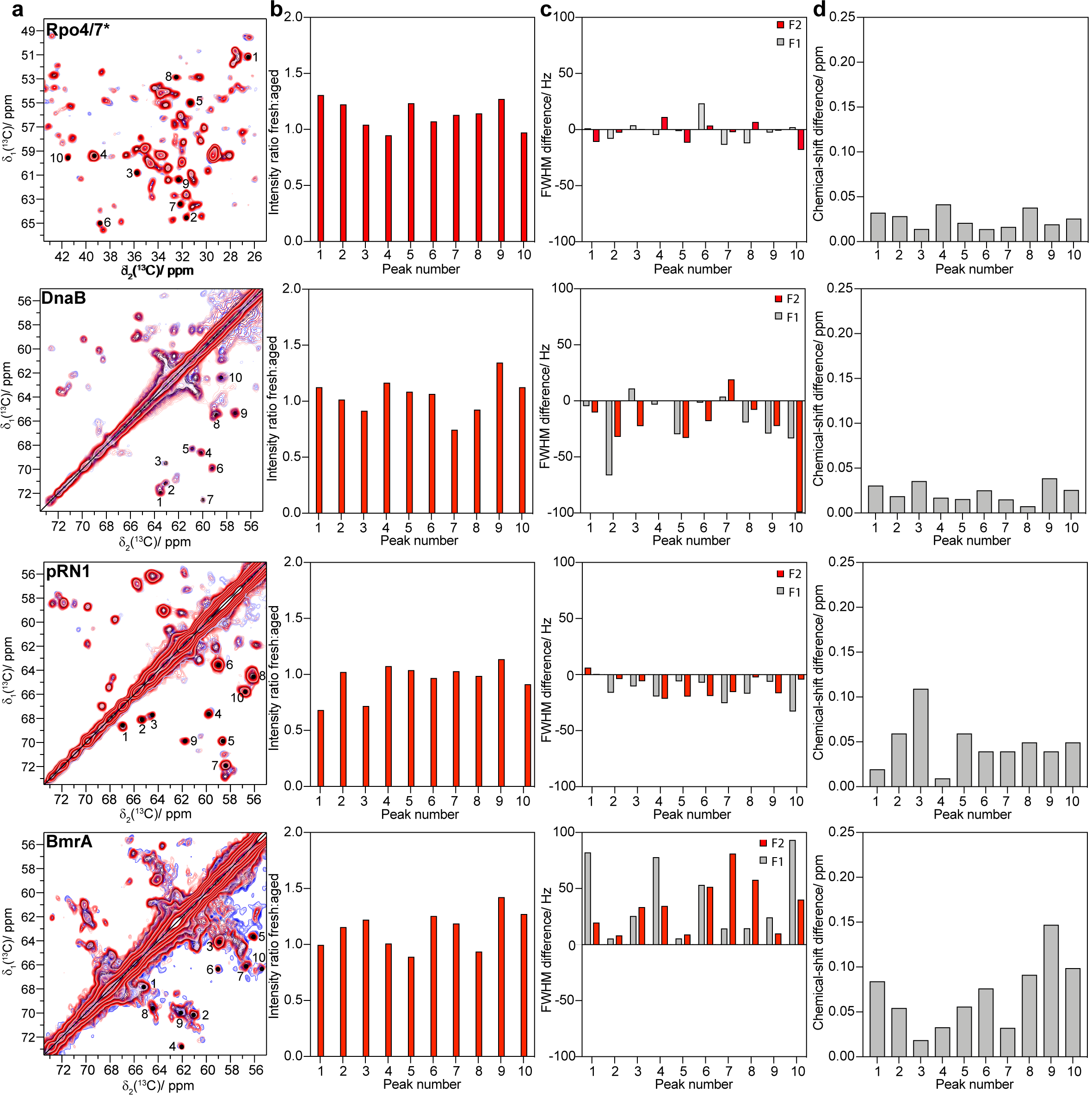
Figure S2:** *Analysis of representative peaks of 20 ms ^13^C-^13^C DARR correlation spectra for fresh and aged samples of the four proteins studied.* **a** Overlay of fingerprints of 20 ms ^13^C-^13^C DARR spectra highlighting the peaks used for the analysis. **b** Comparison of peak intensities, the peak of the aged sample is normalized to one. **c** Differences of the full-widths at half maximum (FWHM) in the F2 and F1 dimensions. **d** Chemical-shift difference calculated according to $\sqrt{\frac{1}{2}\left( {\Delta\delta\left( F1 \right)}^{2}+{\Delta\delta\left( F2 \right)}^{2} \right)}$.
